# Assessment of patient-reported symptom and psychological distress after neoadjuvant chemo-immunotherapy and lung resection for non-small cell lung cancer

**DOI:** 10.1093/icvts/ivag003

**Published:** 2026-01-08

**Authors:** Cecilia Pompili, Javeria Tariq, Pooja Bhatnagar, Nick Brown, Nilanjan Chaudhuri, Katy Clarke, Sun Fei, Kevin Franks, Joshil Lodhia, Richard Milton, Marco Nardini, Kostas Papagiannopoulos, Peter Tcherveniakov, Elaine Teh, Alessandro Brunelli

**Affiliations:** Institute for Clinical & Applied Health Research, Hull University, Hull HU6 7RU, United Kingdom; Department of Thoracic Surgery, St. James’s University Hospital, Leeds Teaching Hospital Trust, Leeds LS2 9DA, United Kingdom; Department of Oncology, St. James’s University Hospital, Leeds teaching Hospital, Leeds LS2 9DA, United Kingdom; Department of Oncology, Calderdale and Huddersfield, NHS Trust, HD3 3EA, United Kingdom; Department of Thoracic Surgery, St. James’s University Hospital, Leeds Teaching Hospital Trust, Leeds LS2 9DA, United Kingdom; Department of Oncology, St. James’s University Hospital, Leeds teaching Hospital, Leeds LS2 9DA, United Kingdom; Department of Oncology, St. James’s University Hospital, Leeds teaching Hospital, Leeds LS2 9DA, United Kingdom; Department of Oncology, St. James’s University Hospital, Leeds teaching Hospital, Leeds LS2 9DA, United Kingdom; Department of Thoracic Surgery, St. James’s University Hospital, Leeds Teaching Hospital Trust, Leeds LS2 9DA, United Kingdom; Department of Thoracic Surgery, St. James’s University Hospital, Leeds Teaching Hospital Trust, Leeds LS2 9DA, United Kingdom; Department of Thoracic Surgery, St. James’s University Hospital, Leeds Teaching Hospital Trust, Leeds LS2 9DA, United Kingdom; Department of Thoracic Surgery, St. James’s University Hospital, Leeds Teaching Hospital Trust, Leeds LS2 9DA, United Kingdom; Department of Thoracic Surgery, St. James’s University Hospital, Leeds Teaching Hospital Trust, Leeds LS2 9DA, United Kingdom; Department of Thoracic Surgery, St. James’s University Hospital, Leeds Teaching Hospital Trust, Leeds LS2 9DA, United Kingdom; Department of Thoracic Surgery, St. James’s University Hospital, Leeds Teaching Hospital Trust, Leeds LS2 9DA, United Kingdom

**Keywords:** lung cancer, quality of life, symptoms, immunotherapy, neoadjuvant therapy, surgery

## Abstract

**Objectives:**

Neoadjuvant chemo-immunotherapy is associated with oncologic benefits in patients undergoing resection for locally advanced non-small cell lung cancer (NSCLC). We assessed patient-reported physical and psychological symptoms following neoadjuvant chemo-immunotherapy and surgery compared to stage-equivalent patients who were operated during the same period without neodjuvant treatment.

**Methods:**

All consecutive patients submitted to lung resection for clinical stage II and III NSCLC between March 2023 and December 2024 and alive at the time of the interview were approached for the study. Their patient-reported symptoms were assessed using the Non-Small Cell Lung Cancer Symptom Assessment Questionnaire (NSCLC-SAQ) and their psychological distress was assessed using the Hospital Anxiety and Depression Scale (HADS).

**Results:**

Of the 138 patients initially screened, 82 completed the survey. Median time from surgery to the interview was 13.9 months. There was no difference in total NSCLC-SAQ score between patients undergoing upfront surgery (S) and surgery after neoadjuvant chemo-immunotherapy (CT-IO) (*P* = .64). Chemo-immunotherapy was not independently associated with total NSCLC-SAQ score after multivariable regression analysis. The average anxiety and depression scores were also similar between the 2 groups. Finally, a similar proportion of patients in the 2 groups reported to have symptoms similar or better than before starting treatment.

**Conclusions:**

Our findings show in a real clinical practice setting that neoadjuvant chemo-immunotherapy is not negatively associated with patient-reported physical or psychological symptoms in the medium to long-term follow-up compared to surgery alone. These results can be used as information tool during patients’ counselling.

## Graphical abstract

**Figure:**
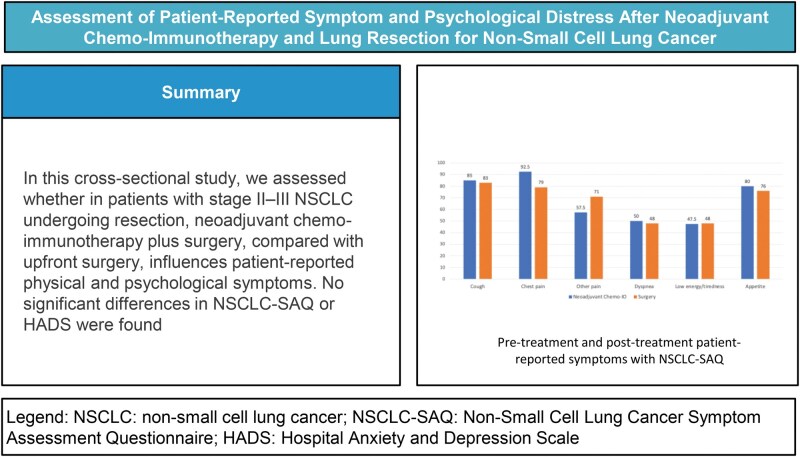

## INTRODUCTION

Recent advances in the multimodal treatment of lung cancer have significantly transformed the therapeutic landscape of this aggressive disease. In particular, the survival benefits associated with various immunotherapy-based regimens have led to a redefinition of treatment pathways, in which surgery now plays an increasingly central role.^1–3^

With the emergence of perioperative strategies^4^—as opposed to neoadjuvant-only approaches—there is growing interest in integrating systemic therapies both before and after surgery. This shift is driven not only by improved oncologic outcomes, but also by the overall tolerability of these regimens. As such, gaining insight into how patients perceive the tolerability of these therapies from their own perspective has become essential to support shared decision-making and personalized treatment planning.

There is still limited evidence on how patients experience these treatments^5^—particularly in the postoperative period and how post-operative complications and long term surgery-related sequelae may interfere with the start of the adjuvant therapy. Understanding the patient-reported impact of these novel therapies is critical, as certain individuals may be at higher risk of postoperative decline.

The objective of this study was to assess patient reported physical and psychological symptoms after undergoing surgery following neoadjuvant chemotherapy and nivolumab compared to stage-equivalent patients who were operated during the same period without neodjuvant treatment.

## PATIENTS AND METHODS

This study was approved by the Leeds Teaching Hospitals Information Governance Department which requested verbal consent from the participating subjects. All patients gave their consents to participate to the telephonic interview and for their responses to be analysed. The consent was collected at the beginning of the telephonic interview. The survey included several questions related to this study (see below) and additional clinical questions about patient recovery beyond the scope of this study. The survey required 5-10 minutes for completion and was conducted by trained staff following a standardized script. Responses were recorded in real-time and entered into a secure database for analysis.

The protocol was reviewed by the Institutional Review Board (Research and Innovation Department) of Leeds Teaching Hospitals and considered as service evaluation, not requiring therefore further review from the research ethics committee. This was a cross-sectional study conducted during the month of March 2025.

All consecutive patients submitted to lung resection for clinical stage II and III Non-Small Cell Lung Cancer (NSCLC) between March 2023 and December 2024 and alive at the time of the interview were initially considered eligible for the study.

Clinical staging was based on the 8th edition of the tumour-node-metastasis staging system. All lung cancer patients were discussed at a multidisciplinary team meeting where the indication for surgery was agreed. They were operated by certified thoracic surgeons and managed in a dedicated general thoracic surgery unit. After surgery pathologic results were discussed in a multidisciplinary team meeting where further management or surveillance was agreed.

### Patient-reported symptoms assessment

Patient-reported symptoms were assessed using the NSCLC Symptom Assessment Questionnaire (SAQ), a validated instrument developed in accordance with FDA guidance for the evaluation of patient-reported outcomes in oncology trials.^6^^,^^7^

The NSCLC-SAQ consists of 7 items that assess the following 5 NSCLC symptom concepts: cough, pain, dyspnoea, fatigue, and poor appetite.

All items have a recall period of the previous 7 days and 5-point response scale ranging from 0 (“not at all”) to 4 (“very severe”) or from 0 (“never”) to 4 (“always”) to measure attributes of symptom intensity or frequency, respectively. The total NSCLC-SAQ score is the sum of the 5 domains and ranges from 0 to 20. Higher scores indicate more severe symptoms or worse symptom burden. The NSCLC-SAQ has demonstrated content validity, construct validity, and internal consistency in previous clinical studies. The questions are detailed in the **Appendix**.

Patients were then asked to grade their current symptoms compared to those experienced prior to the beginning of treatment based on their recollection. To this purpose each individual SAQ symptom scale was graded as better now, same as before, somewhat worse than before, definitely worse than before, extremely worse than before treatment.

### Psychological distress assessment

Psychological distress was assessed using the Hospital Anxiety and Depression Scale (HADS),^8^ a validated 14-item self-report questionnaire specifically designed to detect symptoms of anxiety and depression in medically ill patients, while minimizing the influence of somatic symptoms. The HADS comprises 2 subscales: anxiety (HADS-A) and depression (HADS-D), each including 7 items scored on a 4-point Likert scale (0-3), with total scores ranging from 0 to 21 per subscale. Higher scores indicate greater levels of psychological distress. The HADS has been widely used in oncological populations to evaluate emotional well-being.

### Statistical analysis

The general characteristics along with NSCLC-SAQ scores and HADS of patients with and without neoadjuvant chemo-immunotherapy were compared. Numeric variables with normal distribution were compared using the unpaired Student’s t-test, and those without normal distribution were compared using the Mann–Whitney test. Categorical variables were compared using the Chi square test or Fisher’s exact test (whenever the count in any of the cells was < 10).

Variables for the analysis were extracted from an Institutional prospectively maintained database. All variables were complete.

The following variables were used for comparison and entered into a multivariable regression analysis to assess the independent association of neoadjuvant chemo-immunotherapy with NSCLC-SAQ total score: age, sex, forced expiratory volume in 1 second (FEV1), carbon monoxide lung diffusion capacity (DLCO), presence of coronary artery disease (CAD), Charlson’s Comorbidity Index (CCI), ECOG performance status, extent of surgery (pneumonectomy as opposed to lesser resection), surgical access (open as opposed to minimally invasive), clinical stage and follow-up time (days from surgery to survey date).

In addition, a logistic regression analysis was performed to identify any factor associated with a definitive worse deterioration of current symptom status compared to pretreatment status (based on patient recollection). For this purpose, a definitive worse status was defined as any of the NSCLC-SAQ scale being judged definitively worse or extremely worse than before treatment by the patients.

All tests were performed using the Stata 15.0 statistical software (Stata Corp, College Station, TX, United States).

## RESULTS

A total of 138 patients were initially screened for eligibility and contact (*n* = 62 with neoadjuvant platinum-based chemotherapy and nivolumab [CT-IO], *n* = 76 with surgery upfront [S]). Of these, 13 patients had deceased at the time of contact (*n* = 8 CT-IO, *n* = 5 S), leaving 125 patients eligible for telephone outreach.

**Figure 1.**
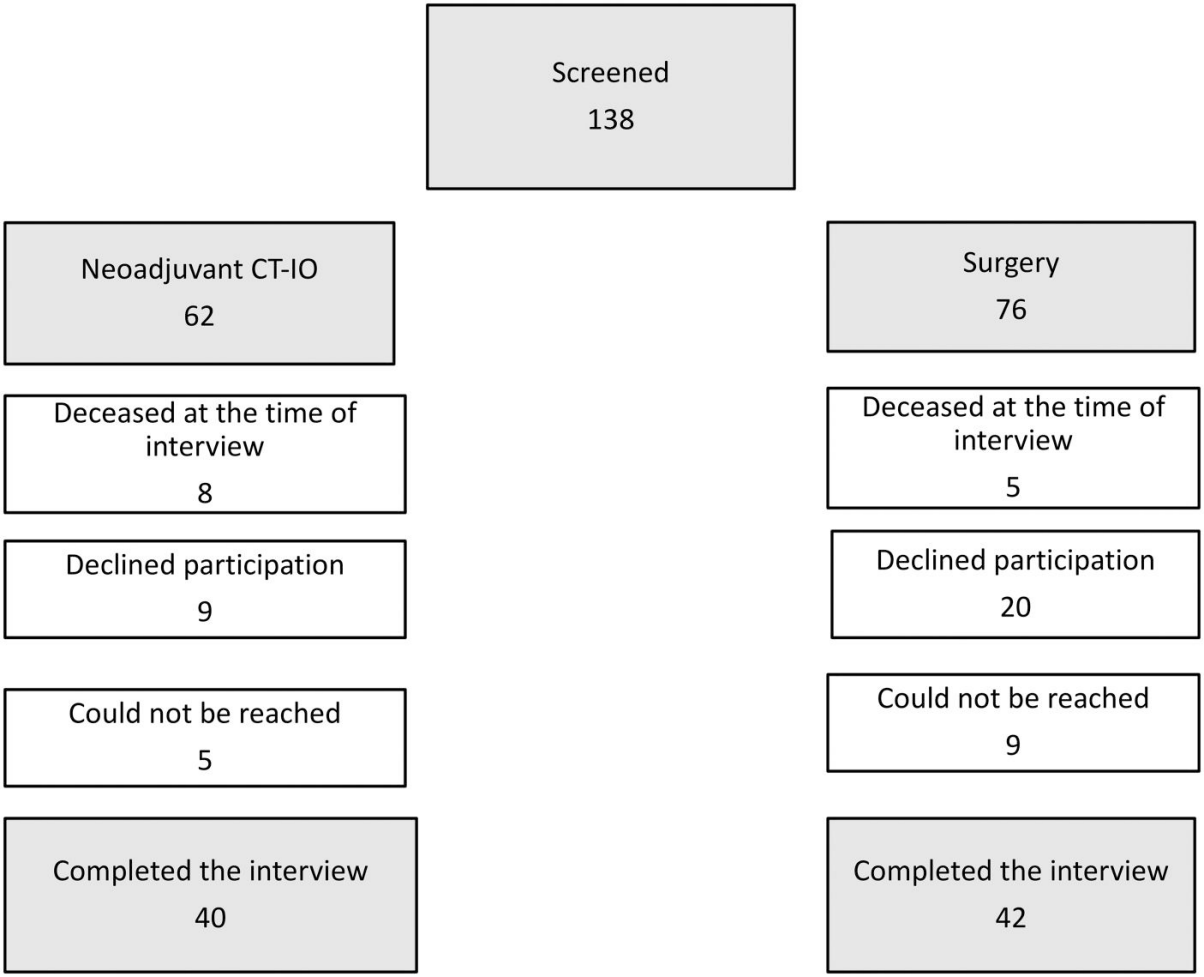
Patient Selection Flowchart.

Among those contacted, 29 declined participations (*n* = 9 CT-IO, *n* = 20 S), and 14 could not be reached despite repeated attempts (*n* = 5 CT-IO, *n* = 9 S). A total of 96 patients provided informed consent (*n* = 45 CT-IO, *n* = 51 S), and 82 completed the interview and were included in the final analysis (*n* = 40 CT-IO, *n* = 42 S) (**Figure 1**).

The most frequent causes for not starting neoadjuvant chemo-immunotherapy were lack of sufficient tissue for molecular testing/tissue diagnosis/staging (15 patients), underlying co-morbidities/frailty (10 patients), presence of actionable genetic alterations (5 patients) and patient choice (5 patients) (see **Appendix Table A1**).

More patients undergoing neoadjuvant chemo-immunotherapy were diagnosed at clinical stage IIIA (21 vs 7) or IIIB (5 vs 0), whereas the non-neoadjuvant group had a higher number of stage II patients (14 vs 35) (*P* < .001).

All patients in the CT-IO group received 3 cycles of platinum-based chemotherapy and nivolumab except 4 patients who had their regimen stopped after 2 cycles for side effects.

The median time from surgery to interview was 416 days (IQR 284-547). It was 325 days (IQR 222-325) in the CT-IO group and 470 days (323-586) in the S group.

The clinical characteristics of the patients included in the analysis are shown in **Table 1**. The 2 groups looked substantially similar except for a younger age and more males in the IO group.

**Table 1.** Characteristics of the Patients Included in the Study

Variables	CT-IO (n.40)	Surgery upfront (n.42)	*P*-value
Age	63.7 (10)	70 (8.5)	.0067
Males (*n*, %)	25 (63%)	17 (40%)	.046
BMI (kg/m^2^)	27.0 (4.3)	27.2 (3.9)	.87
FEV1%	88.2 (19.2)	93.6 (21.0)	.12
DLCO%	74.8 (15.8)	81.2 (20.8)	.19
CAD (*n*, %)	3 (7.5%)	4 (9.5%)	.86
Diabetes (*n*,%)	6 (15%)	5 (12%)	.75
CCI > 1 (*n*, %)	16 (40%)	9 (21%)	.093
Thoracotomy access (as opposed to minimally invasive) (*n*, %)	7 (17%)	7 (17%)	.92
Pneumonectomy (*n*, %)	3 (7.5%)	4 (9.5%)	1
Postop cardiopulmonary Complications (*n*, %)	14 (35%)	12 (29%)	.53
LOS (days)	4.9 (2.2)	5.5 (2.7)	.23

Results are expressed as mean and standard deviations for numeric variables or as count and percentages for categorical variables.

Abbreviations: BMI, body mass index; CAD, coronary artery disease; CCI, Charlson’s co-morbidity index; CT-IO, neoadjuvant chemo-immunotherapy; DLCO, carbon monoxide lung diffusion capacity; FEV1, forced expiratory volume in one second; LOS, postoperative length of stay.

The most frequent procedure was lobectomy (33, 83% in the CT-IO group and 37, 88% in the S one), there were 4 and 1 bilobectomies, respectively.

The incidence of cardiopulmonary complications and the length of stay was similar in the 2 groups (**Table 1**).

Thirteen patients in the S group received adjuvant systemic treatment after surgery (11 chemotherapy alone, 2 Osimertinib, 1 chemo-immunotherapy). None in the CT-IO group received adjuvant systemic treatment.

### Patient-reported outcomes

The average NSCLC-SAQ total score was 6.71 (SD 2.9) in the CT-IO group and 6.57 (SD 3.8) in the S one, *P* = .64.

To eliminate the potential confounder of adjuvant therapy, we repeated the comparison excluding those patients who received any form of adjuvant systemic treatment in the S group. The average NSCLC-SAQ total score in the patients who did not receive adjuvant treatment in the S group was 6.55 (SD 3.8) which remained not different from the average score in the CT-IO group, *P* = .84.

Table A2 shows the comparison of the individual scales between the two groups expressing symptoms experienced during the last 7 days from the interview.

We were unable to find any significant differences between the 2 groups for each scale.

**Figure 2.**
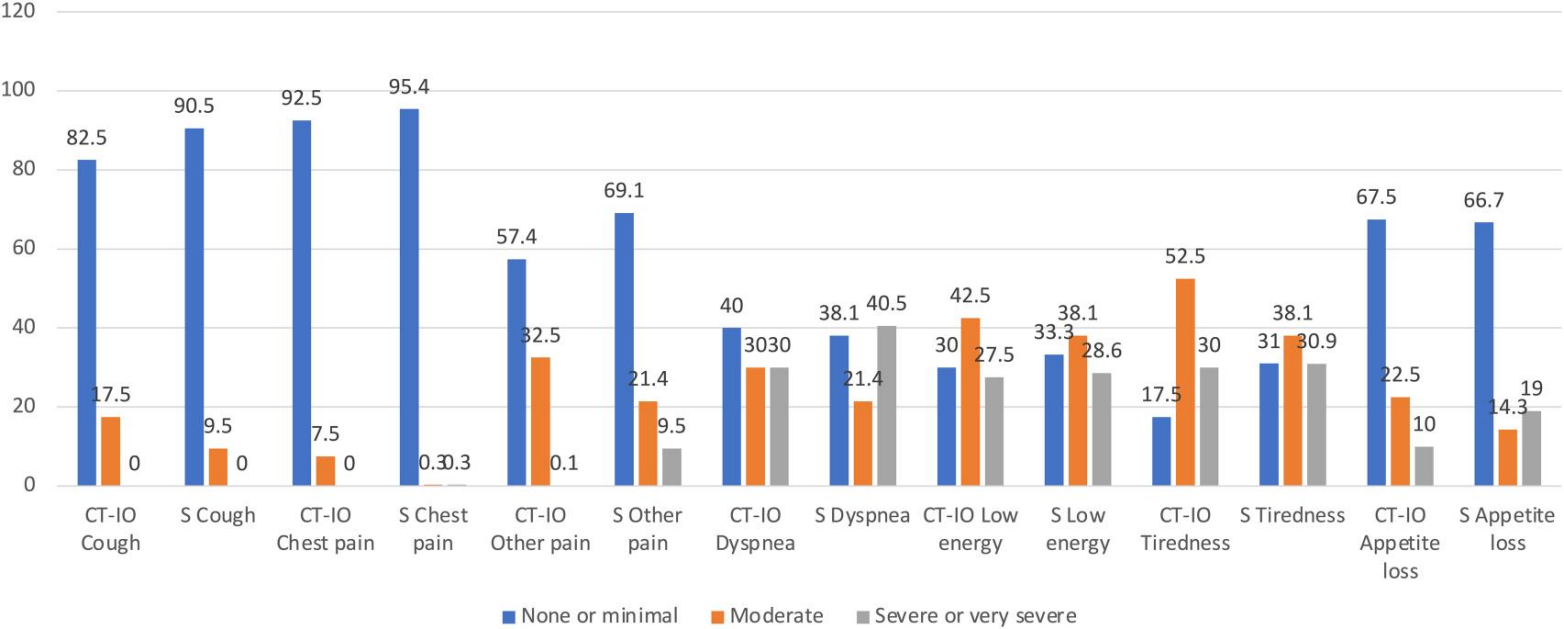
Proportion of Patients in Each Treatment Group Reporting to Have None or Minimal, Moderate, or Severe or Very Severe Symptom Levels for Each SAQ Scale.

**Figure 2** shows the percentages of patients stating to have none or minimal, moderate or severe or very severe symptoms in each group.

After adjusting for multiple confounders using a multivariable regression analysis, we were not able to find neodjuvant chemo-immunotherapy associated with the total SAQ score (regression coefficient 0.71, 95% CI −1.2-2.6, *P* = .45) (**Appendix Table A3**).

In the surgical group, there was no difference in total SAQ between patients who received adjuvant treatment and those who did not (6.55 vs 6.61, *P* = .97).

The average anxiety score was 4.6 (SD 4.1) in the neoadjuvant CT-IO group and 4.4 (SD 3.8) in the S one, *P* = .91. Only 3 patients in each group reported abnormal anxiety levels (score > 10), *P* = 1

The average depression score was 5.0 (SD 4.0) in the neoadjuvant CT-IO group and 4.3 (SD 3.4) in the S one, *P* = .51. Only 1 patient in the CT-IO group and 2 in the S group reported abnormal depression level (score > 10), *P* = 1.

### Exploratory analysis on perceived difference with baseline symptoms

Most patients in both groups (85% in CT-IO and 83% in S) reported that their cough is now better or same as before the start of the treatment. Similarly, they reported their chest pain to be same or better than before treatment (93% CT-IO group vs 79% in S).

About shortness of breath during usual activities, 50% of patients in the CT-IO group and 52% in the S one stated that this symptom is now worse than before treatment with 23% and 29%, respectively reporting it as definitely or extremely worse. Similar trend applied to experiencing low energy level and easy tiredness (**Table 2**). Forty-three percent of patients in each group (17 in the CT-IO group and 18 in the S group) stated that at least one symptom was definitely or extremely worse than before treatment (*P* = .97).

**Table 2.** Comparison of SAQ Scales Compared to the Status Prior to Start the Treatment According to Patient Recollection (Neoadjuvant Chemo-Immunotherapy Group n. 40, Surgery Upfront Group n. 42)

	Better now		Same as before		Somewhat worse than before		Definitely worse than before		Extremely worse than before		*P* value
	CT-IO	S	CT-IO	S	CT-IO	S	CT-IO	S	CT-IO	S	
Cough	17	15	17	20	5	6	1	1	0	0	.91
Chest pain	11	9	26	24	2	7	1	1	0	1	.37
Other pain	5	5	18	25	10	5	3	7	4	0	.083
Dyspnoea	7	10	13	10	11	10	7	8	2	4	.82
Low energy/tiredness	6	6	13	14	15	11	5	9	1	2	.73
Appetite	9	5	23	27	6	6	1	4	1	0	.39

Results are expressed as absolute number of patients in each group.

Abbreviations: CT-IO, neoadjuvant chemo-immunotherapy; S, surgery upfront.

**Figure 3.**
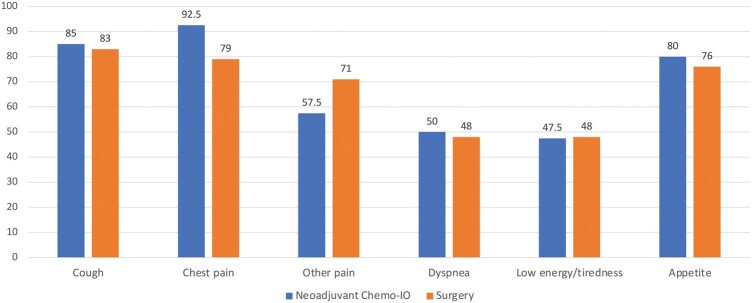
Proportion of Patients in Each Treatment Group Reporting to Have SAQ Symptoms Levels Better or Same as Before Starting the Treatment.

A similar proportion of patients in the 2 groups reported to have symptoms similar or better than before starting treatment (**Figure 3**).

Logistic regression analysis was not able to find neoadjuvant chemo-immunotherapy as an independent factor associated with a definite worse symptom status compared to pretreatment (OR 2.28, 95% CI 0.7-7.5, *P* = .17) (**Appendix Table A4**).

## DISCUSSION

Our findings show no significant differences in patient-reported symptoms or psychological distress following surgery between patients who received neoadjuvant therapy and those who did not. Notably, this study was conducted in a real-world clinical setting, providing novel insights into the patient experience in routine care.

Landmark trials in these settings have recently reported similar Patient-reported Outcomes (PROMs) after neoadjuvant therapy, complementing the remarkable clinical advantages on this new immunotherapy-based treatment regimens.^4^^,^^9^^,^^10^

However, trials of novel treatments in early-stage disease often failed to report robust HRQoL outcomes with many results either omitted from primary publications or presented only as secondary abstracts, limiting their visibility and impact within the scientific community.

The Checkmate-77T trial,^4^ using the same questionnaire, reported not clinically meaningful overall change from baseline in the NSCLC-SAQ score between patients who received perioperative immunotherapy plus chemotherapy compared to those receiving only neoadjuvant chemotherapy. However, this trial did not compare these symptoms with patients who were submitted to surgery upfront.

Similar results were confirmed by the KEYNOTE-671 trial^3^ that showed no between-group differences in the least-squares mean change from baseline in the neoadjuvant or adjuvant phase for patient reported HRQoL, physical functioning, role functioning, dyspnoea, cough, or chest pain. However, these trials were assessing postoperative patient-reported data only in the context of adjuvant cycle (active treatment of placebo), limiting the interpretation of these results being more affected by surgery or its complications.

Checkmate-816 is the only trial^11^ showing so far long-term maintenance of HRQoL with neoadjuvant nivolumab + chemo in patients with NSCLC undergoing surgery detailing post-surgery recovery of HRQoL according to surgical access and extent of resection, but with a limited peri-surgical assessment around 2 months after surgery.

Our analysis in a real-world setting showed that adding neoadjuvant immuno-chemotherapy is not adversely affecting the postoperative lung cancer related symptoms reported by the patients. Post-operative anxiety and depression were also similar in the 2 groups at a medium-long term follow-up.

Interestingly, 31% of patients in the surgical group received adjuvant systemic treatment after surgery, but this treatment did not appear to affect their reported symptoms compared to those who did not receive any systemic treatment after surgery.

Although the current evidence reports immunotherapy to be well tolerated, some of the Immune-related adverse events (irAEs) have the potential to affect patients’ lives in different way, especially as there are still significant knowledge gaps in our understanding of irAEs.^12^ These challenges are also related to the potential intersection of this toxicity with surgical morbidity and long term HRQoL. This analysis reports for the first time the potential effect of these agents on the psycho-physical wellbeing of resectable patients comparing their symptoms to the ones who did not receive neoadjuvant chemo-immunotherapy.

Despite we were not able to find statistically significant association between the administration of neoadjuvant chemo-immunotherapy and deterioration of symptoms following surgery, caution must be exercised when interpreting our results given the small sample size. The moderately high odd ratio (OR 2.28) may still indicate a clinically meaningful effect of chemo-immunotherapy on the outcome. However, the wide confidence intervals show large variability, and therefore the true effect is highly uncertain. This precludes to draw definitive conclusion on this potential clinical association, warranting future investigations.

This study is subject to several limitations inherent to its methodology.

We used a telephone interview to collect PRO data. While conducted using a validated and structured approach, this method may introduce biases such as social desirability bias, recall bias, and interviewer bias. The absence of visual cues may also affect the depth of responses. These findings should ideally be validated through direct patient-reported data collection methods, such as electronic completion.

A second limitation is the choice of symptom-focused questionnaire. We cannot exclude the possibility that using tools incorporating functional outcomes, such as the EORTC LC29,^13^ which was specifically developed for lung cancer multimodality treatments, might yield different results.

Another limitation lies in the cross-sectional design of our study. While this approach facilitates higher response rates, it does not allow for the longitudinal analysis of inter-patient changes over time, which could provide more granular insights. Additionally, the absence of a baseline patient-reported assessment means that comparisons rely on patient recall of their preoperative state, introducing the potential for recall bias, response-shift bias (where a patient’s internal standards or perception of symptoms may change post-treatment), and retrospective bias. These factors may affect the accuracy of self-reported preoperative experiences. Future studies with baseline PROMs assessment and longitudinal analysis will be needed to confirm reliability of our findings.

Another important limitation which is common to longitudinal studies focusing on PROMs is the inherent survivorship bias since inclusion required being alive at contact to receive the interview. A sensitivity analysis was performed to compare the characteristics of interviewed patients and those who were screened but not interviewed. We did not find any relevant difference (Table A5).

This study is not a randomized trial and treatment allocation was based on clinical selection. As such the 2 treatment groups may have inherent differences which may have affected the results. In the attempt to minimize this problem, we also performed multivariable regression and logistic regression analyses adjusting for possible confounders. We cannot rule out however that unrecorded or unknown factors may have played a role.

## CONCLUSION

This study, conducted in a real-world, single-centre setting, explores how patient-reported tolerability of neoadjuvant chemo-immunotherapy compares to that of patients undergoing upfront surgery. We were not able to find significant differences in symptoms or psychological distress between the 2 groups. These findings support the notion that, although neoadjuvant chemo-immunotherapy may involve treatment-related toxicities, these do not appear to negatively impact PROMs in the medium to long-term follow-up. Considering the numerous limitations and especially the absence of baseline reported PROMs and small sample size, our results can be used as information tool during patients’ counselling about this multimodal management.

## Supplementary Material

ivag003_Supplementary_Data

## Data Availability

The data underlying this article will be shared on reasonable request to the corresponding author.
