# Supplementary material for: Assessment of patient-reported symptom and psychological distress after neoadjuvant chemo-immunotherapy and lung resection for non-small cell lung cancer
Source: Interdiscip Cardiovasc Thorac Surg. 2026 Jan 8;41(1):ivag003. doi: 10.1093/icvts/ivag003 (PMC12821362; doi:10.1093/icvts/ivag003)
Supplement: ivag003_Supplementary_Data [file ivag003_supplementary_data.docx]

Appendix

Non-small cell lung cancer Symptom Assessment Questionnaire (NASCLC-SAQ)

For each of the following questions, please choose the one response that best describes your experience over the last 7 days.

1. How would you rate your coughing at its worst over the last 7 days?

No Coughing at All, Mild Coughing, Moderate Coughing, Severe Coughing, Very Severe Coughing

2. How would you rate the worst pain in your chest over the last 7 days?

No Pain at All, Mild Pain, Moderate Pain , Severe Pain, Very Severe Pain

3. How would you rate the worst pain in areas other than your chest over the last 7 days?

No Pain at All, Mild Pain, Moderate Pain, Severe Pain, Very Severe Pain

4. How often did you feel short of breath during usual activities over the last 7 days?

Never, Rarely, Sometimes, Often, Always

5. How often did you have low energy over the last 7 days?

Never, Rarely, Sometimes, Often, Always

6. How often did you tire easily over the last 7 days?

Never, Rarely, Sometimes, Often, Always

7. How often did you have a poor appetite over the last 7 days?

Never, Rarely, Sometimes, Often, Always

Table A 1: Reasons for not starting neoadjuvant chemo-immunotherapy in the non-IO patients included in the analysis (total no. 42)

| Reason | Number |
| --- | --- |
| Lack of tissue for molecular testing or diagnosis | 15 |
| AGA | 5 |
| Underlying co-morbidities/frailty | 6 |
| Patients’ choice | 5 |
| Underlying immune disease | 4 |
| Pulmonary fibrosis | 2 |
| Logistics/MDT decision | 2 |
| Unclear stage at diagnosis | 1 |
| Mucoepidermoid histology unlikely to benefit. | 1 |
| Synchronous primary | 1 |

AGA: actionable genetic alterations; MDT: multidisciplinary team.

Table A2: Comparison of the individual scales between the two groups expressing symptoms experienced during the last 7 days from the interview

For each scale scores ranges from 0 (from no symptoms or never experienced) to 4 (very severe or always experienced).

| **NSCLC-SAC scales** | **Neoadjuvant CT-IO (n.40)** | **Surgery upfront (n.42)** | **p value** |
| --- | --- | --- | --- |
| How much cough | 0.83 (0.1) | 0.57 (0.1) | 0.096 |
| How much chest pain | 0.35 (0.1) | 0.50 (0.1) | 0.21 |
| How much pain in other areas | 1.25 (1.0) | 0.95 (1.1) | 0.13 |
| How often dyspnea during usual activities | 1.8 (1.1) | 1.9 (1.4) | 0.78 |
| How often low energy | 1.85 (1.1) | 1.86 (1.1) | 0.98 |
| How often easily tiring | 2.07 (1.0) | 1.95 (1.2) | 0.58 |
| How often poor appetite | 0.85 (1.1) | 1.0 (1.5) | 0.99 |

Results are expressed as means and standard deviations. CT-IO: neoadjuvant chemo-immunotherapy

Table A3: results of the multivariable regression analysis to identify factors associated with total NSCLC-SAQ score (dependent variable)

| Variables | Coefficients | p value | 95%CI |
| --- | --- | --- | --- |
| Age | -0.44 | 0.33 | -0.13-0.04 |
| Sex (male) | -0.92 | 0.30 | -2.67-0.83 |
| FEV1% | -0.01 | 0.58 | -0.06-0.03 |
| DLCO% | 0.02 | 0.34 | -0.03-0.08 |
| Neoadjuvant chemo-IO | 0.71 | 0.45 | -1.16-2.59 |
| Pneumonectomy | 0.28 | 0.87 | -3.14-3.71 |
| CCI | -0.19 | 0.86 | -2.23-1.86 |
| Performance Status | 2.31 | 0.53 | -5.02-9.63 |
| Minimally invasive access | -0.56 | 0.38 | -1.18-0.69 |
| Coronary artery disease | -0.39 | 0.80 | -3.42-2.64 |
| Clinical stage III | -0.28 | 0.77 | -2.20-1.64 |
| Follow up time | 0.004 | 0.23 | -0.002-0.009 |

CCI: Charlson’s Comorbidity Index; FEV1: forced expiratory volume in one second; DLCO: carbon monoxide lung diffusion capacity. Follow up time: the time elapsed from surgery to the interview.

Table A4: results of the logistic regression analysis to identify factors associated with a definitely or extremely worse deterioration of symptoms compared to pre-treatment status

| Variables | OR | p value | 95%CI |
| --- | --- | --- | --- |
| Age | 1.02 | 0.35 | 0.97-1.09 |
| Sex (male) | 1.20 | 0.74 | 0.41-3.57 |
| FEV1% | 1.01 | 0.65 | 0.98-1.04 |
| DLCO% | 0.99 | 0.97 | 0.97-1.03 |
| Neoadjuvant chemo-IO | 2.28 | 0.17 | 0.69-7.54 |
| Pneumonectomy | 0.12 | 0.13 | 0.01-1.80 |
| CCI | 1.53 | 0.53 | 0.39-5.97 |
| Minimally invasive access | 0.17 | 0.063 | 0.03-1.09 |
| Coronary artery disease | 0.68 | 0.72 | 0.08-5.48 |
| Clinical stage III | 0.63 | 0.47 | 0.18-2.22 |
| Follow up time | 1.00 | 0.004 | 1.00-1.01 |

CCI: Charlson’s Comorbidity Index; FEV1: forced expiratory volume in one second; DLCO: carbon monoxide lung diffusion capacity. Follow up time: the time elapsed from surgery to the interview.

Table A5: Comparison between interviewed patients and those who were screened but not interviewed (for any cause)

| Variable | Interviewed (82 patients) | Not Interviewed (56 patients) | p-value |
| --- | --- | --- | --- |
| Age | 66.9 (9.9) | 67.2 (10.3) | 0.80 |
| Male sex | 42 (51%) | 28 (50%) | 0.88 |
| FEV1 | 90.9 (20.2) | 89.2 (19.9) | 0.47 |
| DLCO | 78.1 (18.7) | 80.2 (21.3) | 0.58 |
| BMI | 27.1 (4.0) | 28.2 (5.2) | 0.32 |
| CAD | 7 (8.5%) | 5 (8.9%) | 1 |
| Diabetes | 11 (13%) | 9 (16%) | 0.81 |
| CCI>1 | 25 (30%) | 15 (27%) | 0.64 |
| Thoracotomy (as opposed to minimally invasive) | 14 (17%) | 15 (27%) | 0.17 |
| Pneumonectomy | 7 (8.5%) | 3 (5.3%) | 0.74 |

Results are expressed as means and standard deviations for numeric variables or count and percentages for categorical ones. CCI: Charlson’s Comorbidity Index; BMI: body mass index; CAD: coronary artery disease; FEV1: forced expiratory volume in one second; DLCO: carbon monoxide lung diffusion capacity
